# Social influence increases the value and consumption of alcohol in the laboratory

**DOI:** 10.1111/acer.70115

**Published:** 2025-08-11

**Authors:** Jack Yates, Benjamin Miller, Alazne Arraztio Cordoba, Jasmine Grace Warren, Michael Batterley, Jessica Catherine Gay, Abigail K. Rose, Carl A. Roberts, Andrew Jones

**Affiliations:** ^1^ University of Liverpool Liverpool UK; ^2^ Universidad Loyola Andalucía Córdoba Spain; ^3^ Liverpool John Moores University Liverpool UK

**Keywords:** addiction, ad libitum, alcohol, behavioral economics, value‐based decision making

## Abstract

**Background:**

Previous research has demonstrated the perceived value of alcohol is transient in hypothetical social and environmental contexts. This study sought to further expand on this by examining whether the social influence of a confederate and the physical environment could be manipulated to influence the value of alcohol and ad libitum alcohol consumption, and thus provide support for the role of value as a mechanism underlying alcohol use.

**Method:**

A total of 140 (90 female, Mean age = 25.81, SD = 14.20, Mean AUDIT = 11.51, SD = 5.38) participants completed a between‐subjects 2 (environment: bar labortaory vs. standard unadorned) × 2 (social influence: positive appraisal vs. negative appraisal) design in which they completed a brief assessment of alcohol demand, a concurrent choice task, and a visual analogue scale measuring alcohol value, following a limited drinking session with a confederate in one of two laboratory settings, and then completed an ad libitum bogus taste test.

**Results:**

Social influence had a significant effect on intensity index of demand (*F* (1,133) = 4.74, *p* = 0.031, ηp^2^ = 0.03) and on ad libitum consumption (*F* (1,135) = 7.60, *p* = 0.007, *ηp* ^*2*^ = 0.05) with positive appraisal having greater intensity scores (Mean = 4.34, SD = 2.80) compared with the negative appraisal (Mean = 3.39, SD = 2.23) and more alcohol consumed (Mean = 221.07 mL, SD = 121.76 vs. Mean = 164.71 mL, SD = 111.80). The intensity index also mediated the relationship between social influence and ad libitum consumption (B = 10.40, 95% Bootstrapped CIs = 0.34 to 23.59). There were no significant main effects of environment and no interactions between social influence and environment.

**Conclusion:**

These findings suggest alcohol value is sensitive to social influence. Increased value as a result of positive alcohol appraisals by others had a significant effect on ad libitum consumption and that the intensity index of demand mediated the relationship.

## Graphical abstract

Social influence shapes the perceived value of alcohol, driving consumption. This study examined how positive and negative social appraisals by a confederate impact alcohol's relative value and ad libitum consumption. We found that positive appraisals increased the intensity index of demand as well as consumption, with intensity mediating the relationship between social influence and consumption. This suggests that greater relative value, influenced by positive social cues, drives higher consumption and that relative value is potentially the mechanism responsible.

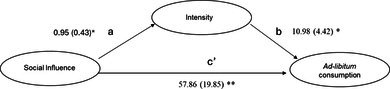

## INTRODUCTION

Each year, 132.6 million healthy years (disability‐adjusted life years) are lost to alcohol use, while alcohol consumption accounts for 5.3% of all deaths (World Health Organization, 2018). In England alone, the estimated socioeconomic costs of alcohol consumption are around £21 billion per year (Public Health England, 2016). Irrespective of a multitude of harms, many individuals still consume alcohol at harmful levels. Several cognitive mechanisms are hypothesized to play a role in alcohol use, such as inhibitory control (Yücel et al., 2019). However, empirical evidence suggests these mechanisms explain small amounts of variance at best (Baines & Jones, 2021; Smith & Mattick, 2018). While comparing variance explained across studies and behavioral mechanisms may not reflect clinical/practical significance and should be interpreted cautiously, it does suggest that these are unlikely the primary psychological mechanism(s) that underpin consumption and dependence (Hogarth, 2020).

Recently, there has been a shift in attempting to understand alcohol use by applying behavioral economic approaches (Bickel et al., 2014; Hogarth & Field, 2020; Tucker et al., 2021). Constructs such as *demand*—the quantitative measure of the relationship between cost and the attainment of a commodity (e.g., alcohol)—have been reliably associated with both quantity and frequency of alcohol consumption (Murphy & MacKillop, 2006) and are predictive of brief treatment outcomes (MacKillop & Murphy, 2007; Murphy et al., 2015). However, these behavioral economic approaches take a “molar” perspective in that they attempt to understand the distribution of behavior by looking at overall patterns (Vuchinich et al., 2023) and as such do not examine the underlying cognitive processes which contribute to individual decisions to use a substance.

Value‐based decision making (VBDM) is a theoretical framework that can be used to address this gap by attempting to model the internal processes that occur in the lead‐up to a decision to drink alcohol being made, based upon the idea that people act in accordance with what is valued most to them in that moment (Berkman et al., 2017; Levy & Glimcher, 2012; Rangel et al., 2008). Berkman et al. (2017) proposed that decisions to obtain one commodity over another (e.g., alcohol over a soft drink) are determined by a greater relative value of one option (alcohol) over competing alternatives (soft drinks). More specifically, the value of each possible option is derived from integrating various gains and costs (tangible, social, and self‐related) collectively known as *value inputs*, which can vary by person, context, and time. More recently, Field et al. (2020) proposed the idea that the VBDM framework can be readily applied to addiction and alcohol use in particular, with the greater relative value of alcohol leading to decisions to drink.

There has been increased focus on testing the application of VBDM to alcohol use. One approach has been to apply drift‐diffusion models to two‐alternative forced‐choice tasks measuring value for alcohol (Copeland et al., 2023). Drift‐diffusion models are a form of computation modeling that parametrizes the decision‐making process (Myers et al., 2022), which provides indices of evidence accumulation (known as drift‐rate)—which is thought to represent the speed and direction of internal decisions to choose an option (e.g., alcohol). Using this paradigm alongside simple behavioral economic measures for alcohol demand, Copeland et al. (2023) compared 60 current heavy drinkers and 60 former heavy but now moderate drinkers, and hypothesized that compared to heavy drinkers, moderate drinkers will have low alcohol demand, lower evidence accumulation rates for alcohol, and greater evidence accumulation rates for soft drinks. However, their findings suggested there were no differences in evidence accumulation between the groups, but there was some evidence for increased behavioral economic measures. Copeland et al. (2024) also used drift‐diffusion models to investigate whether alcohol could successfully be devalued using videos that emphasized the positives (increasing the value) or the negatives (decreasing the value) of alcohol. They demonstrated the devaluation of alcohol led to increased evidence accumulation for soft drinks, but not reduced evidence accumulation for alcohol. Notably, this difference might not have been identified in tasks designed to measure relative value (such as concurrent choice tasks: Hardy et al., 2018), which provide a percentage choice for one commodity over another.

These findings suggest that drift‐diffusion techniques may be better at modeling internal processes. However, they offer an “indirect” approach (Dora et al., 2024). Specifically, individuals do not make relative choices (e.g., alcohol vs. food), but rather choose between high‐value alcohol versus low‐value alcohol. Similarly, participants in these tasks are reminded of their value ratings, which might transiently impact these ratings irrespective of value‐based inputs (Copeland et al., 2022). As such, it is difficult to model decisions based on relative value of one commodity over another without direct comparisons, such as concurrent choice tasks (Dora et al., 2024). However, when using alternate tasks that do make direct comparisons (e.g., concurrent choice tasks), it is difficult to model the internal processes and therefore, it is more likely relative value that is being indexed.

There is a similar yet distinct model of behavioral economic —Contextualized Reinforcer Pathology (Acuff et al., 2023)—that considers the role of relative value and incorporates wider choice context. Unlike the traditional reinforcer models of behavioral economics, the contextualize reinforcer pathology model posits that a drug's reinforcing value is not an innate quality of a drug but is instead critically determined by characteristics of the choice environment (Acuff et al., 2023) similar to Berkman et al.'s (2017) VBDM. Unlike VBDM, Contextualized Reinforcer Pathology is still a “molar” perspective; however, both are recent perspectives that consider the wider choice environment and posit different contextual factors or “value inputs” in Berkman et al. (2017) affect the relative value of a substance and thus the decision to consume said substance.

To fully understand alcohol use as a potential consequence of value‐based decision making, it is important to consider the roles of specific *value inputs* identified in theoretical models such as Berkman et al. (2017). Few studies have tested these inputs on alcohol use (in the absence of indices of value). For instance, previous research has examined the roles of context and social influence on ad libitum consumption and demonstrates that when with a confederate who is drinking alcohol (whether they are known or unknown to the participant), participants will consume more alcohol (Dallas et al., 2014; Larsen et al., 2009, 2010, 2012; Robinson et al., 2016). Additionally, when also considering environmental contexts, Monk and Heim (2013, 2014) found that typical drinking locations such as real and simulated pubs, bars, and clubs were associated with heightened outcome expectancies when compared with other settings, which, if viewed through a value‐based framework, could be taken to indicate higher value. However, these studies did not directly measure changes in value as a proposed mechanism.

To date, there are three studies that have investigated the relationship between social context and alcohol demand. Acuff, Soltis and Murphy (2020) had participants complete two modified (one explicitly social and one explicitly solitary) alcohol purchase tasks (Murphy & MacKillop, 2006). They found that participants reported significantly greater demand in the social variant compared with the solitary variant, suggesting the presence of peers increases alcohol demand and therefore alcohol's relative value. Moreover, Yates et al. (2023) sought to establish the transiency of alcohol value as a result of context and social influence. They presented various hypothetical drinking scenarios (drinking alone vs. drinking with friends who were also drinking versus drinking with friends by trying to “cut down” versus drinking with friends who were not drinking) to participants prior to them completing the brief assessment of alcohol demand (BAAD). They demonstrated increased value for alcohol when drinking with friends who were also drinking, suggesting a role of social influence on value. In a second study, four categories of images were presented in a 2 (environment: bar vs. house) × 2 (social influence: enjoy vs. not enjoy) design and measured value using a concurrent choice task (CCT) and visual analogue scale (VAS). This study demonstrated no effect on value. However, a limitation of both studies is that they were conducted online and used hypothetical scenarios, which have no real‐world consequences for participants (Klein & Hilbig, 2019). Furthermore, as it was impossible to directly measure subsequent alcohol consumption in these studies, the causal pathway from value input > value > alcohol use cannot be tested, although one further study did begin to model this pathway (Acuff, MacKillop, & Murphy, 2020). Using social network analysis, Acuff and colleagues found that social network alcohol density significantly predicted alcohol misuse as well as alcohol demand, further suggesting the presence of peers, in particular peers consuming alcohol, increases alcohol consumption. Moreover, that there was an indirect effect on alcohol misuse through alcohol demand demonstrated the potential pathway through value input > value > alcohol use. However, as with the previous studies, no in‐person alcohol consumption was measured.

As such, the aims of this study were to assess the impact of the study environment and the social influence of a confederate (our hypothesized value inputs) on (1) the subjective value of alcohol, (2) the amount of alcohol consumed, and (3) whether the subjective value of alcohol mediates the relationship between the value inputs and alcohol use.

To do this, this study adopted a 2 (environment: bar laboratory vs. standard unadorned laboratory) × 2 (social influence: positive appraisal vs. negative appraisal) design in which participants completed proxy measures of value following a limited drinking session with a confederate in one of two laboratory settings and then completed an ad libitum bogus taste test. We hypothesized that (H1)—a positive appraisal of alcohol by a confederate (social influence) would lead to a significant increase in subjective value and ad libitum alcohol consumption, compared to a negative appraisal of alcohol by a confederate; (H2)—a seminaturalistic environment that would promote drinking behavior (a bar laboratory) would lead to a significant increase in subjective value and ad libitum alcohol consumption, compared to a standard unadorned environment; (H3)—a significant interaction between social influence and environment, in that the combined impact of positive social influence and a seminaturalistic drinking environment would lead to the highest subjective value of alcohol and ad libitum alcohol consumption; and (H4)—ALCOHOL use disorder symptom severity (as measured by the Alcohol Use Disorders Identification Test), ad libitum consumption, and craving (as measured by the Alcohol Urge Questionnaire) will be predictors of greater ascription of value to alcohol.

This study was preregistered at https://osf.io/z893r/, data and analysis scripts can be found here https://osf.io/z893r/files/osfstorage.

## METHOD

### Participants

One‐hundred forty participants (90 female, Mean age = 25.81, SD = 14.20, Mean AUDIT score = 11.51, SD = 5.38) were recruited from a student population using a university research participation scheme and the wider community using posters and social media (Facebook) posts. Data outcomes for ad libitum alcohol use and value measures were not recorded for one participant who dropped out, and data for two participants on value measures were lost due to computer error. Inclusion criteria include participants aged 18+ years and consuming alcohol regularly (10+ units per week). Exclusion criteria include previous or current diagnosis of alcohol use disorder or being on medication contraindicated to alcohol. A priori sample size calculation using G*power analysis determined *n* = 128 would be the sample size to achieve 80% power (Cohen, 1988) to detect a moderate effect size (*f* = 0.25) for the main effects of social influence and environment on value at a significance level of 0.05. This study was granted ethical approval by the host university's ethics committee (REF: 10989, 03‐03‐2022) and all participants provided informed consent. Data collection took place between April 2022 and July 2024.

### Materials

#### Alcohol Use Disorder Identification (AUDIT) (Saunders et al., 1993)

The AUDIT is a scale assessing quantity and frequency of alcohol use as well as behavior and consequences associated with drinking. The AUDIT contains 10 items and demonstrated good internal consistency in this sample (ωt = 0.82) and has been shown to have excellent validity (Daeppen et al., 2000).

#### Alcohol Urge Questionnaire (AUQ) (Bohn et al., 1995)

The AUQ is an 8‐item questionnaire providing an index of acute craving. The AUQ had excellent internal consistency (ωt = 0.95) within this sample and has been validated for the real‐time measurement of alcohol craving in human laboratory research (MacKillop, 2006).

#### Subjective Intoxication Scales (SIS) (Duka et al., 1998)

The SIS consisted of six 5‐point Likert scales (strongly disagree to strongly agree) which assessed subjective feelings of “lightheaded,” “irritable,” “stimulated,” “alert,” “relaxed,” and “contented.” Previous studies have shown the SIS is sensitive to alcohol intoxication (Jackson et al., 2001; Rose & Duka, 2006).

### Value‐based measures

#### Brief assessment of alcohol demand (BAAD) (Owens et al., 2015)

The BAAD is a 3‐item scale assessing the most widely used indices of alcohol demand: Intensity, Omax, and Breakpoint. Intensity is the maximum consumption at no cost, and is measured using the question “If drinks were free, how many would you have?” Responses to this question were given in number of drinks and choices ranged from 0 to 10+ in 1 drink increments. Omax is the greatest expenditure an individual is willing to spend on alcohol across prices, and measured using the question “What is the maximum total amount you would spend on drinking during that drinking occasion?” Responses to this question were given in pounds and choices ranged from £0 to £30+. Breakpoint is the price at which consumption of alcohol is suppressed measured using the question “What is the maximum amount you would pay for a single drink?”. Responses to this question were given in pounds and choices ranged from £0 to £15+ in £1 increments. The BAAD has been found to produce comparable results to those obtained with the full Alcohol Purchase Task (Merrill & Aston, 2020; Owens et al., 2015).

#### Visual analogue scale (VAS) (Hayes & Patterson, 1921)

The VAS had anchor points at 0 and 100. Participants were asked to rate “how much they value” the alcoholic drink they had just consumed by clicking their cursor at a point along the line. VASs have been reliably used to measure momentary value in previous research (Chen et al., 2016).

#### Concurrent choice task (CCT)

Participants were presented with 40 two‐alternative‐forced choice trials of alcohol and soft drink images in which they were required to press a key corresponding to either image to win “points.” Each choice had only a 50% chance of yielding a point to increase switching between rewards (Rose et al., 2013). As concurrent choice tasks reflect goal‐directed behavior toward a drug (Hogarth, 2020), they are considered to be indicative of the relative value of the drug versus the alternative reward (Hardy et al., 2018; Hogarth & Field, 2020; Moeller et al., 2013).

Over the 40 trials, the left/right location of the alcohol and nonalcohol stimulus was counterbalanced. The alcohol and soft drink images were taken from the Amsterdam Beverage Picture Set (ABPS; Pronk et al., 2015). Concurrent choice tasks have been validated as a measure of the relative value of one commodity over another (Hogarth & Field, 2020).

### Procedure

Participants were randomly allocated to one of two locations (standard unadorned laboratory or the seminaturalistic bar laboratory, see supplementary materials Figures S1 and S2 for visualizations) and one of two social influence conditions (positive appraisal of the alcohol by a confederate or negative appraisal of the alcohol by a confederate). The seminaturalistic bar laboratory is a purpose‐built laboratory designed to increase the ecological validity of alcohol research by imitating a typical British pub environment including beer pumps, shelves of spirits with optic mounts, posters, and other alcoholic advertisements and has been validated as a setting to promote alcohol use in previous studies (Field & Jones, 2017; Jones et al., 2013; McGrath et al., 2016). The standard unadorned laboratory was approximately the same size, furnished with tables and chairs. Participants and the confederate were initially breathalyzed (randomization to condition occurred prior to participant attendance) and separated using a room divider under the pretense of completing the initial questionnaires anonymously.

The confederate was unknown to the participant and was instructed to attend the experiment meeting point at the same time the participant was given so that the researcher could take them both into the laboratory together. The confederate was one of a possible 8 (demographics: 29F, 27F, 20F, 26F, 32F, 20M, 24M, 29M). Participant and confederate gender was not matched as previous research, both experimentally and observationally, has shown there are no gender differences in drinking imitation for participant gender or confederate gender (Larsen et al., 2010, 2012), and confederates were chosen for each session based on their availability at the time. Sensitivity analyses indicated that the effect of confederate appraisal on alcohol consumption was more pronounced when the confederate was of the opposite gender to the participant, suggesting a potential moderating role of interpersonal gender dynamics (see Supplementary Materials for exploratory factor of confederate/participant gender matching in Appendix S1).

The participant then completed the AUDIT, first AUQ, and first SIS. Participant and confederate were then brought together for an initial limited drinking session in which both were presented with a small half‐pint (285 mL) glass of either beer (4.5% ABV) or cider (4.5% ABV) depending on preference (confederate drink is alcohol‐free alternative) and asked to drink as much or as little as they choose in 5‐min and rate the taste of the drink using a 9‐point Likert scale (anchors: Like Extremely to Dislike Extremely). The confederate was given specific instructions depending on the condition. During the positive appraisal condition, the confederate was instructed to rate the drink as “like very much” and tell the participant they were doing so. They were also instructed to say to the participant that they “really liked” the drink, and also instructed to drink the majority of the beverage so it was visible to the participant. For the negative appraisal condition the confederate was given the opposite instructions to rate as “dislike very much” and to tell the participant they “really disliked” the drink, they drank only a small amount of the beverage. Aside from this, confederates were not given an exact script to follow as this would not represent a realistic context (Larsen et al., 2009) and may have increased participant awareness of the study aims if the conversation felt unnatural.

**FIGURE 1:**
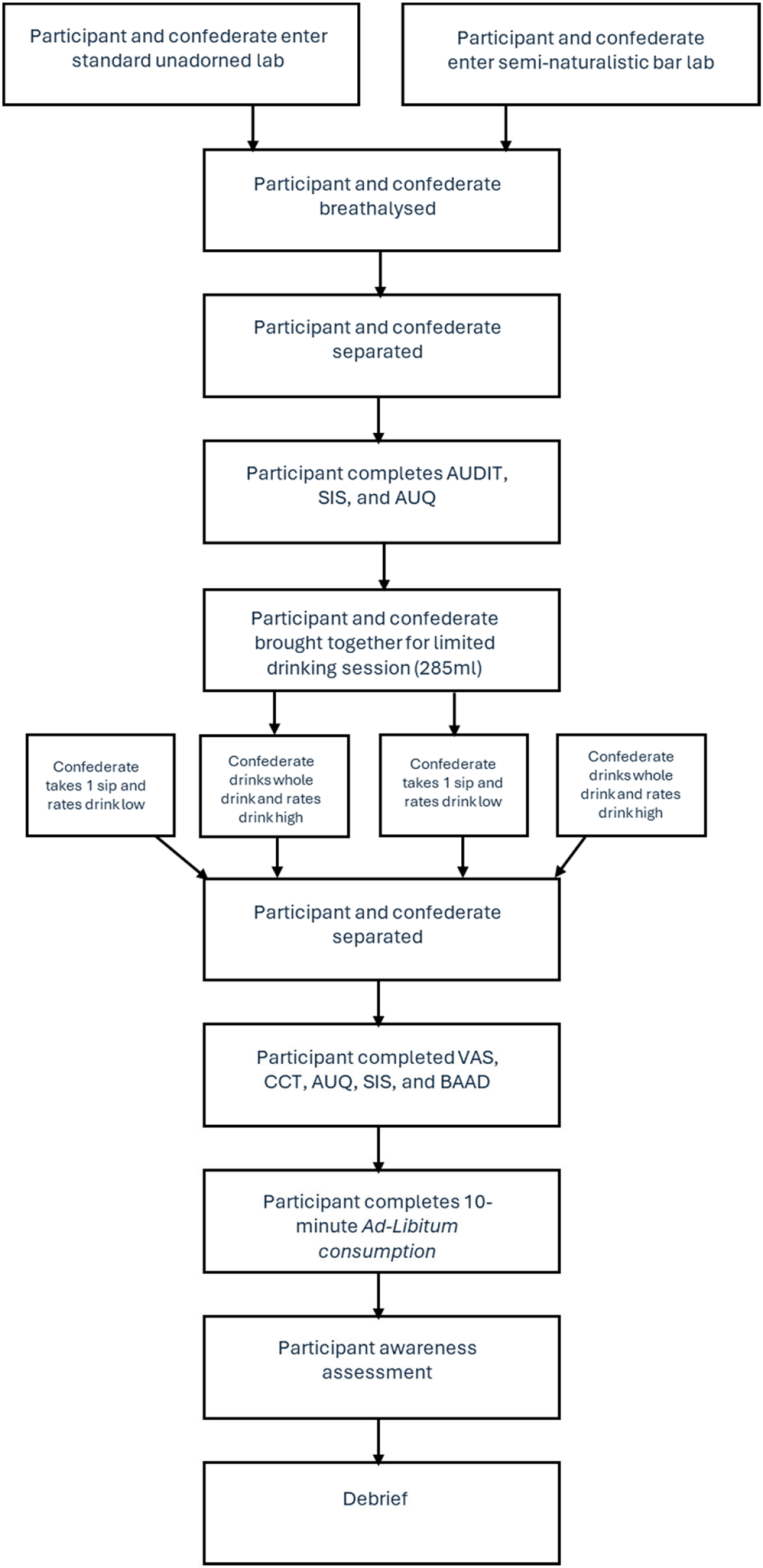
Procedure flow chart. AUDIT, Alcohol Use Disorder Identification Test; AUQ, Alcohol Urge Questionnaire; BAAD, Brief Assessment of Alcohol Demand; CCT, Concurrent Choice Task; SIS, Subjective Intoxication Scale; VAS, Visual Analogue Scale.

Following the first drinking session, participants and confederates were again separated behind dividers, and the participant completed the VAS, the CCT, a second AUQ and SIS, and the BAAD. Participants were then presented with 1 pint (568 mL) of the same drink they chose earlier for 10 min of ad libitum consumption and again asked to drink as much or as little as they wanted while rating the taste on the 9‐point Likert scale. Participants had originally been led to believe that we were interested in the effect of glass size on perceived taste as a cover story to reduce awareness of the measurement of consumption. The participant and confederate would remain separate for the ad libitum drinking to prevent any mimicry effects that might have been observed. However, participants would also believe the confederate was drinking behind the divider. Finally, participants were asked to report what they believed the aims of the study were (to assess awareness of any deception). One participant correctly guessed the aims of the study, and as such, analyses were performed including and excluding them, and the results were found to be unchanged. Participants were then debriefed and breathalyzed again to ensure participants did not leave the laboratory over 0.17 mg/L BrAc (Figure 1).

### Data analysis

Data analysis was conducted using R studio, with the “dplyr” (Wickham et al., 2023), “ggstatsplot” (Patil, 2021), “WRS2” (Mair & Wilcox, 2020), “psych” (Revelle, 2020), and “lavaan” (Rosseel, 2012) packages. Ad libitum alcohol consumption (in mL), the three BAAD indices, VAS rating, and percentage alcohol choice in the concurrent choice task (number of times alcohol images were chosen/total trials × 100) were the dependent variable for repeated measures 2 (Social influence: Positive vs. Negative) × 2 (Environment: Bar laboratory vs. Standard unadorned laboratory) ANOVAs. Given some deviations from normality for these variables, we also conducted sensitivity analyses using robust ANOVAS based on medians and also ANOVAS based on 10% trimmed means (a 10% trimmed mean cuts off 10% at the lower end and 10% the higher end of the distribution: see Mair & Wilcox, 2020). This analysis did not substantially influence the primary results (no change in *p*‐values from significant to nonsignificant or vice versa); therefore, we retained results from nonrobust 2 × 2 ANOVAS in line with our preregistration.

Correlations were also performed between the value proxies and ad libitum consumption. Additionally, linear regressions were conducted to determine whether ad libitum consumption, craving change (as assessed by the difference between time 1 and 2 in the alcohol urge questionnaire) and AUDIT score predicted any of the value proxies. Finally, an exploratory mediation analysis was conducted to examine the effect of social influence (positive vs. negative appraisal) on alcohol consumption, with intensity serving as a mediator. Bootstrapping with 5000 samples was used to estimate confidence intervals for the effects.

## RESULTS

### Participant characteristics

Table 1 summarises the participant characteristics of the current sample split by condition.

**TABLE 1:** Demographic information of the participants, split by experimental condition.

Experimental condition	Mean age (SD)	Mean AUDIT (SD)	Gender
Bar laboratory/Positive Appraisal	26.37 (17.00)	11.64 (4.94)	23 female, 12 male
Bar laboratory/Negative appraisal	24.80 (14.07)	11.57 (5.75)	23 female, 12 male
Standard unadorned laboratory/Positive appraisal	28.20 (14.43)	10.71 (5.76)	22 female, 13 male
Standard unadorned laboratory/Negative appraisal	23.89 (10.86)	12.15 (5.14)	22 female, 13 male
Total	25.81 (14.20)	11.51 (5.38)	90 female, 50 male

Table 2 shows the means and standard deviations across conditions.

**TABLE 2:** Means and standard deviations of proxy measures of value across conditions.

	Bar laboratory/positive appraisal	Bar laboratory/negative appraisal	Standard unadorned laboratory/positive appraisal	Standard unadorned laboratory/negative appraisal
Intensity	Mean = 4.33, SD = 2.59	Mean = 3.46, SD = 2.31	Mean = 4.34, SD = 3.04	Mean = 3.32, SD = 2.18
Omax	Mean = 15.00, SD =8.20	Mean = 12.71, SD = 8.52	Mean = 17.43, SD = 10.74	Mean = 13.97, SD = 9.44
Breakpoint	Mean = 4.12, SD = 1.75	Mean = 3.54, SD = 1.09	Mean = 4.14, SD = 1.46	Mean = 3.76, SD = 1.54
CCT	Mean = 51.59%, SD = 22.94%	Mean = 52.21%, SD = 22.09%	Mean = 47.93%, SD = 20.10%	Mean = 45.52%, SD = 18.90%
VAS	Mean = 50.61, SD = 26.43	Mean = 38.85, SD = 25.75	Mean = 53.85, SD = 25.32	Mean = 51.36, SD = 22.23

*Note*: Intensity = number of drinks if free; Omax = amount willing to spend on single drink in £; Breakpoint = total amount willing to spend on drinking occasion in £; CCT = Concurrent Choice Task for alcohol (% indicates alcohol choices);VAS = Visual Analogue Scale (value indicates score on 0 = no value to 100 = high value).

Table 3 demonstrates that the only significant effect found was the main effect of social influence on intensity. Higher intensity scores were found in the confederate positive appraisal condition (Mean = 4.34 SD = 2.80) compared with the negative appraisal condition (Mean = 3.39 SD =2.23, d = 0.38).

**TABLE 3:** ANOVA results for the main effects and interactions of social influence and environment on proxy measures of value.

	Social influence	Environment	Social influence x environment
Intensity	*F* (1,133) = 4.74, *p* = 0.031, ηp^2^ = 0.03*	*F* (1,133) = 0.2, *p* = 0.885, ηp^2^ = 0.00	*F* (1,133) =0.027, *p* = 0.870, ηp^2^ = 0.00
Omax	*F* (1,133) = 3.38, *p* = 0.068, ηp^2^ = 0.02	*F* (1,133) = 1.34, *p* = 0.249, ηp^2^ = 0.01	*F* (1,133) =0.14, *p* = 0.713, ηp^2^ = 0.00
Breakpoint	*F* (1,133) = 3.64, *p* = 0.059, ηp^2^ = 0.03	*F* (1,133) = 0.24, *p* = 0.630, ηp^2^ = 0.00	*F* (1,133) =0.16, *p* = 0.692, ηp^2^ = 0.00
CCT	*F* (1,133) = 0.05, *p* = 0.826, ηp^2^ = 0.00	*F* (1,133) = 2.08, *p* = 0.151, ηp^2^ = 0.02	*F* (1,133) = 0.18, *p* = 0.674, ηp^2^ = 0.00
VAS	*F* (1,133) = 2.89, *p* = 0.091, ηp^2^ = 0.02	*F* (1,133) = 3.43, *p* = 0.066, ηp^2^ = 0.03	*F* (1,133) = 1.18, *p* = 0.280, ηp^2^ = 0.01

*Note*: Intensity = number of drinks if free; Omax = amount willing to spend on single drink in £; Breakpoint = total amount willing to spend on drinking occasion in £; CCT = Concurrent Choice Task for alcohol (% indicates alcohol choices);VAS = Visual Analogue Scale (value indicates score on 0 = no value to 100 = high value).

* Significant at the *p* < 0.05 level.

#### Ad libitum consumption

**FIGURE 2:**
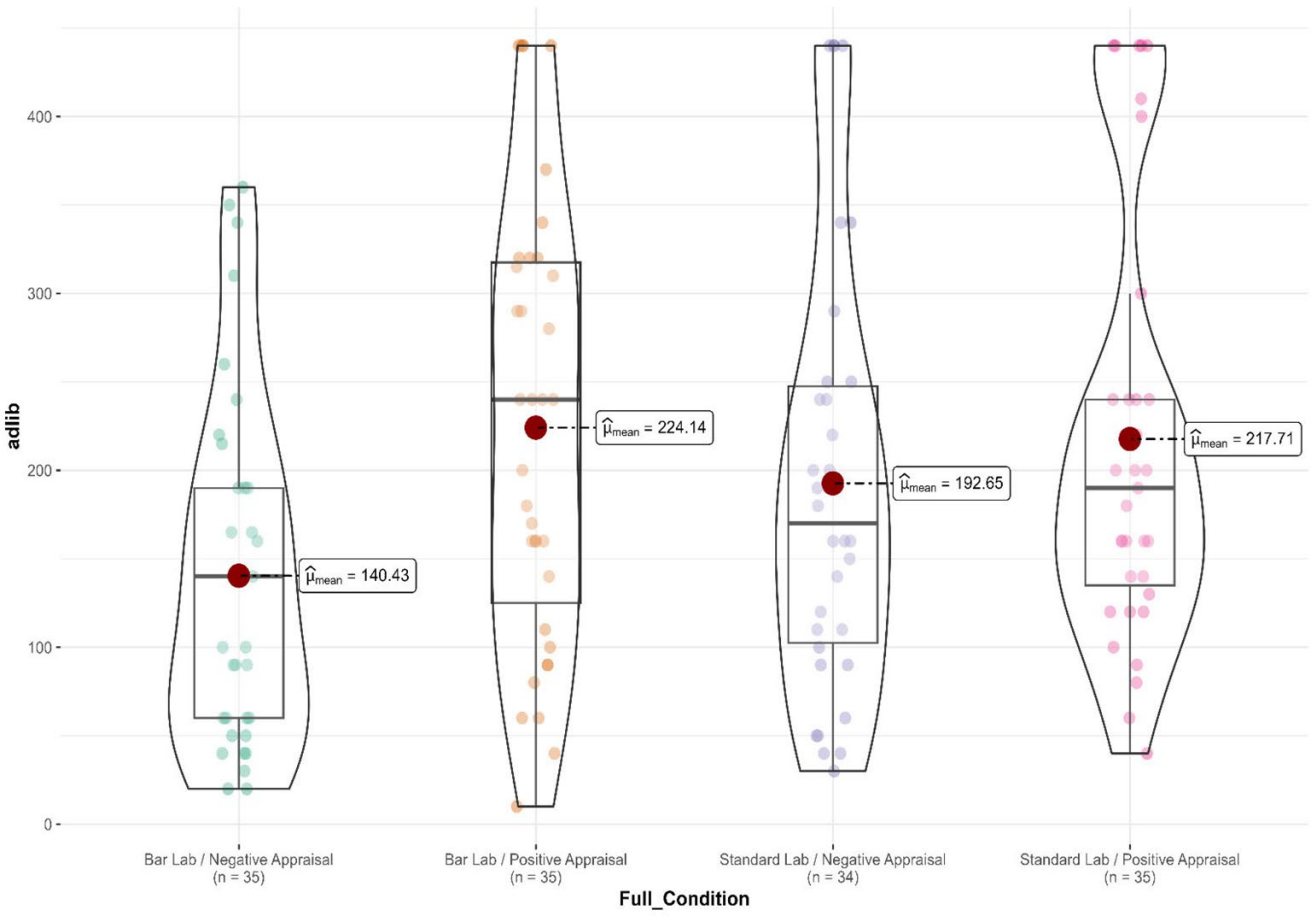
Ad libitum alcohol consumption in milliliters across the 2 (Social Influence: Positive vs. Negative) × 2 (Environment: Bar laboratory vs. Standard unadorned laboratory) groups. There is a main effect of social influence, indicating positive appraisal increased alcohol consumption versus negative appraisal. Large dot in each condition = mean ad libitum consumption in each condition. Small dots = individual participant ad libitum consumption. Shape of plots = visualization of distribution across conditions.

A 2 × 2 between‐subjects ANOVA was conducted to determine the effect of social influence and environment on the amount of alcohol consumed ad libitum. There was a significant main effect of social influence on alcohol consumed (*F* (1,135) = 7.60, *p* = 0.007, *ηp* ^*2*^ = 0.05) with more alcohol consumed in the positive appraisal condition (Mean = 221.07 mL, SD = 121.76) compared to the confederate negative appraisal condition (Mean = 164.71 mL, SD = 111.80: d = 0.48 [95%: 0.14 to 0.81]). However, there was no significant main effect of environment (*F* (1,135) = 1.30, *p = 0*.255, *ηp* ^*2*^ = 0.01) and no significant interaction (*F* (1,135) = 2.18, *p = 0*.142, *ηp* ^*2*^ = 0.02) (Figure 2).

#### Value associations

Table 4 shows that intensity and the VAS rating of the alcoholic drink were significantly associated with ad libitum consumption.

**TABLE 4:** Associations between ad libitum alcohol consumption and the proxies for value.

	Intensity	Omax	Breakpoint	CCT	VAS
Ad libitum consumption	*r* = 0.27*	*r* = 0.14	*r* = −0.12	*r* = 0.23	*r* = 0.21*
	*p* = 0.012	*p* = 0.430	*p* = 0.460	*p* = 0.080	*p* = 0.010

* *p* < 0.05.

#### Mediation of intensity between social influence and ad libitum consumption

**FIGURE 3:**
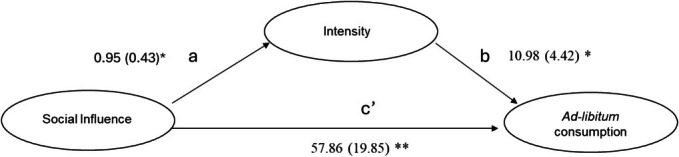
Mediation pathway demonstrated the indirect effect of intensity on the relationship between social influence and ad libitum *consumption*. **p < 0.01; *p < 0.05.

The total effect model revealed that social influence significantly predicted alcohol consumption, *R* ^2^ = 0.058, *F* (1,135) = 8.33, *p* = 0.004, meaning that 5.8% of the variance in alcohol consumption could be explained by social influence and intensity (*B* = 57.86, 95% Bootstrapped CI = 19.25 to 97.29) (Figure 3).

The indirect effect of social influence on alcohol consumption through intensity was also significant (*B* = 10.40, 95% Bootstrapped CIs = 0.34 to 23.59) indicating that intensity significantly mediates the relationship between social influence and alcohol consumption as the bootstrapped confidence interval does not include zero.

#### Value predictors

Linear regressions were performed to determine the predictors of value proxies.

Table 5 shows ad libitum consumption predicted variance in three of the 5 proxy value measures (intensity, CCT, and VAS) and the change in AUQ predicted variance in the intensity index.

**TABLE 5:** Predictors of value proxies in a linear regression, based on alcohol consumption variables.

	Intensity	Omax	Breakpoint	CCT	VAS
Ad libitum	*Β* = 0.01 SE =0.00, CI = −0.01 to 0.00, *p* < 0.001***	*Β* = −0.01, SE = 0.01, CI = −0.02 to 0.01, *p* = 0.194	*Β* = 0.00, SE = 0.00, CI = 0.00 to 0.00, *p* = 0.153	*Β* = −0.04, SE = 0.02, CI = −0.07 to −0.01, *p* = 0.015*	*Β* = −0.04, SE = 0.02, CI = −0.07 to −0.00, *p* = 0.033*
AUDIT	*Β* = 0.07, SE = 0.04, CI = 0.01 to 0.15, *p* = 0.084	*Β* = −0.12, SE = 0.15, CI = −0.42 to 0.18, *p* = 0.425	*Β* = 0.01, SE = 0.02, CI = −0.04 to 0.06, *p* = 0.695	*Β* = −0.15, SE = 0.34, CI = −0.81 to 0.52, *p* = 0.661	*Β* = −0.24, SE = 0.40, CI = ‐1.03 to 0.56, *p* = 0.555
AUQ change	*Β* = 0.08, SE = 0.04, CI = 0.01 to 0.16, *p* = 0.035*	*Β* = 0.21 SE =0.15 CI = ‐0.09 to 0.50 *p* = 0.165	*Β* = 0.02, SE = 0.02, CI = −0.03 to 0.07, *p* = 0.417	*Β* = 0.24, SE = 0.33, CI = −0.42 to 0.89, *p* = 0.478	*Β* = 0.75, SE = 0.40, CI = −0.04 to 1.54, *p* = 0.063

*** *p* < 0.001,

* *p* < 0.05.

## DISCUSSION

The current study sought to examine the effects of social influence and environment on a participant's subjective value of alcohol and the amount of alcohol they consumed ad libitum. We found a main effect of social influence on ad libitum consumption and the intensity index of the BAAD. Greater consumption and higher intensity scores were found when the confederate had a positive appraisal for the alcohol in the limited drinking session compared to when they gave a negative appraisal. We did not, however, find any effects of environment on ad libitum consumption or any of the proxy measures of value, or any interactions between social influence and environment. We also found that only ad libitum consumption predicted variance in intensity; CCT and VAS and AUQ change predicted variance in intensity. As such, we did not fully support any of our preregistered hypotheses. In exploratory mediation, we demonstrated the effects of social influence on ad libitum *consumption* were mediated by intensity, suggesting value does play a mechanistic role in the decision to consume alcohol.

Our finding that social influence had a significant effect on ad libitum consumption is consistent with previous work on the effect of social influence and alcohol consumption (Dallas et al., 2014; Larsen et al., 2009, 2010, 2012; Robinson et al., 2016) in that participants drink more alcohol when the confederate drinks more alcohol and provides a positive appraisal of the drink (however, notably our effect size was somewhat smaller than other studies, which may be possibly explained by having multiple possible confederates). What this study also demonstrates is that this effect persists after the participant and confederate are separated, and this is potentially due to the increased value of the alcohol (based on the intensity index) following the positive appraisal.

Social influence not having a significant effect on omax or breakpoint might be explained by the value naturally being suppressed in laboratory environments regardless of the seminaturalistic setting due to time of day and unrealistic obligations to drink (Larsen et al., 2009). With intensity being consistently the most sensitive index of the BAAD to changes in value (Acuff, MacKillop, & Murphy, 2020; Acuff, Soltis, & Murphy, 2020; Yates et al., 2023), it is possible that intensity is the only demand index sensitive enough to have detected any changes. Intensity is also the only index of demand thought to reflect pure value, irrespective of financial costs (MacKillop et al., 2009). It also may be the case that the omax and breakpoint null findings stem from the use of the BAAD. With the BAAD, omax and breakpoint are derived from single items and not from molar behavioral patterns as they would be in the standard alcohol purchase task (Murphy & MacKillop, 2006; Tucker & Vuchinich, 2015), whereas intensity is always a single item. However, with the CCT and VAS also unable to detect any changes in value, consistent with Yates et al. (2023), there may be a much simpler explanation that the role of alcohol value is smaller than initially thought and there are other unknown mechanisms.

The lack of significant effects of environment on any value proxy or ad libitum consumption is inconsistent with previous research (Monk & Heim, 2013, 2014; Stanesby et al., 2019). Again, this may be partly explained by the natural suppression of drinking behaviors under observation, and the seminaturalistic bar laboratory failing to fully represent a real‐world environment. A replication of the study using a real pub/bar would be an important follow‐up in order to determine whether behaviors are suppressed in the laboratory environments (Gough et al., 2021; Robinson et al., 2015).

There are some more limitations of the current study to be aware of when considering these findings, however. As discussed, the CCT and VAS may not be sensitive enough tasks to detect small changes in value, and a task more suited to detecting these changes and more suited to modeling the internal processes of VBDM, such as the task used in Copeland et al. (2023, 2024), might be better utilized in future studies to expand on the current work. The VBDM task models value using evidence accumulation rates (Ratcliff & McKoon, 2008), which is a somewhat closer representation of how the value process might work according to (Berkman et al., 2017). Additionally, as discussed in the introduction, the tasks used might better represent Contextualized reinforcer pathology.

A further limitation of the current study is that we failed to control for time of day (although all testing began after 12 pm) or day of the week, and epidemiological evidence suggests increased consumption on evenings/weekends (Room et al., 2012; Stanesby et al. 2019). However, although these variables have not been shown to moderate ad libitum drinking in the laboratory (Jones et al., 2013). Additionally, future research could expand on these findings by examining other value inputs that might lead to more robust changes in the value of alcohol in experimental settings such as emotional affect (Dora et al., 2024) and the presence of alcohol‐free alternative activities (Weinsztok et al., 2023). It would also be beneficial to examine how value inputs affect moment‐to‐moment changes in value and how that affects alcohol consumption in the real world using ecological momentary assessment designs such as seen in Motschman et al. (2022).

In conclusion, we demonstrated that social influence has a significant effect on ad libitum consumption and a measure of relative value (intensity), whereby positive appraisal of alcohol by a confederate led to increased alcohol consumption, as well as the subjective value of alcohol (higher intensity score). We also found that intensity significantly mediated the relationship between social influence and ad libitum consumption, suggesting that the relative value of alcohol is at least in part involved in decisions to consume alcohol.

## CONFLICT OF INTEREST STATEMENT

Carl Roberts declares that he has received grant funding from Unilever and has acted as a consultant to pharmaceutical company Beohringer Ingelheim, neither of which are related to the current work. The remaining authors report no conflicting/competing interests and report no financial/personal interests or beliefs that could affect their objectivity.

## Supporting information


Appendix S1

## Data Availability

The data that support the findings of this study are openly available in OSF at https://osf.io/z893r/files/osfstorage.

## References

[acer70115-bib-0001] Acuff, S.F. , MacKillop, J. & Murphy, J.G. (2020) Integrating behavioral economic and social network influences in understanding alcohol misuse in a diverse sample of emerging adults. Alcoholism: Clinical and Experimental Research, 44(7), 1444–1455. 32568458 10.1111/acer.14351 PMC7572548

[acer70115-bib-0002] Acuff, S.F. , MacKillop, J. & Murphy, J.G. (2023) A contextualized reinforcer pathology approach to addiction. Nature Reviews Psychology, 2(5), 309–323. 10.1038/s44159-023-00167-y PMC10028332 37193018

[acer70115-bib-0003] Acuff, S.F. , Soltis, K.E. & Murphy, J.G. (2020) Using demand curves to quantify the reinforcing value of social and solitary drinking. Alcoholism: Clinical and Experimental Research, 44(7), 1497–1507. 32472649 10.1111/acer.14382 PMC7572865

[acer70115-bib-0004] Baines, L. & Jones, A. (2021) The associations between proactive slowing, working memory, alcohol sensitivity, and alcohol use. Journal of Studies on Alcohol and Drugs, 82(1), 142–151. 33573732

[acer70115-bib-0005] Berkman, E.T. , Hutcherson, C.A. , Livingston, J.L. , Kahn, L.E. & Inzlicht, M. (2017) Self‐control as value‐based choice. Current Directions in Psychological Science, 26(5), 422–428. Available from: 10.1177/0963721417704394 29335665 PMC5765996

[acer70115-bib-0006] Bickel, W.K. , Johnson, M.W. , Koffarnus, M.N. , MacKillop, J. & Murphy, J.G. (2014) The behavioral economics of substance use disorders: reinforcement pathologies and their repair. Annual Review of Clinical Psychology, 10, 641–677. Available from: 10.1146/annurev-clinpsy-032813-153724 PMC4501268 24679180

[acer70115-bib-0007] Bohn, M.J. , Krahn, D.D. & Staehler, B.A. (1995) Development and initial validation of a measure of drinking urges in abstinent alcoholics. Alcoholism: Clinical and Experimental Research, 19(3), 600–606. 7573780 10.1111/j.1530-0277.1995.tb01554.x

[acer70115-bib-0008] Chen, Z. , Veling, H. , Dijksterhuis, A. & Holland, R.W. (2016) How does not responding to appetitive stimuli cause devaluation: evaluative conditioning or response inhibition? Journal of Experimental Psychology: General, 145(12), 1687–1701. 27736134 10.1037/xge0000236

[acer70115-bib-0070] Cohen, J. (1988) Statistical power analysis for the behavioral sciences, 2nd edition. Lawrence Erlbaum Associates.

[acer70115-bib-0011] Copeland, A. , Stafford, T. & Field, M. (2022) Methodological issues with value‐based decision‐making (VBDM) tasks: the effect of trial wording on evidence accumulation outputs from the EZ drift‐diffusion model. Cogent Psychology, 9(1), 2079801.

[acer70115-bib-0012] Copeland, A. , Stafford, T. & Field, M. (2024) Value‐based decision‐making in regular alcohol consumers following experimental manipulation of alcohol value. Addictive Behaviors, 156, 108069. 38788454 10.1016/j.addbeh.2024.108069

[acer70115-bib-0010] Copeland, A. , Stafford, T. , Acuff, S.F. , Murphy, J.G. & Field, M. (2023) Behavioral economic and value‐based decision‐making constructs that discriminate current heavy drinkers versus people who reduced their drinking without treatment. Psychology of Addictive Behaviors, 37(1), 132–143. 35901378 10.1037/adb0000873

[acer70115-bib-0013] Daeppen, J.B. , Yersin, B. , Landry, U. , Pécoud, A. & Decrey, H. (2000) Reliability and validity of the alcohol use disorders identification test (AUDIT) imbedded within a general health risk screening questionnaire: results of a survey in 332 primary care patients. Alcoholism: Clinical and Experimental Research, 24(5), 659–665. 10832907

[acer70115-bib-0014] Dallas, R. , Field, M. , Jones, A. , Christiansen, P. , Rose, A. & Robinson, E. (2014) Influenced but unaware: social influence on alcohol drinking among social acquaintances. Alcoholism: Clinical and Experimental Research, 38(5), 1448–1453. 24588229 10.1111/acer.12375

[acer70115-bib-0015] Dora, J. , Kuczynski, A.M. , Schultz, M.E. , Acuff, S.F. , Murphy, J.G. & King, K.M. (2024) An experimental investigation into the effect of negative affect on the behavioral economic demand for alcohol. Psychology of Addictive Behaviors, 38, 409–423. 38190199 10.1037/adb0000984

[acer70115-bib-0016] Duka, T. , Tasker, R. & Stephens, D.N. (1998) Alcohol choice and outcome expectancies in social drinkers. Behavioural Pharmacology, 9(7), 643–653. 9862089 10.1097/00008877-199811000-00019

[acer70115-bib-0018] Field, M. & Jones, A. (2017) Elevated alcohol consumption following alcohol cue exposure is partially mediated by reduced inhibitory control and increased craving. Psychopharmacology, 234, 2979–2988. 28741032 10.1007/s00213-017-4694-6 PMC5591800

[acer70115-bib-0017] Field, M. , Heather, N. , Murphy, J.G. , Stafford, T. , Tucker, J.A. & Witkiewitz, K. (2020) Recovery from addiction: behavioral economics and value‐based decision making. Psychology of Addictive Behaviors, 34(1), 182–193. Available from: 10.1037/adb0000518 31599604

[acer70115-bib-0019] Gough, T. , Haynes, A. , Clarke, K. , Hansell, A. , Kaimkhani, M. , Price, B. et al. (2021) Out of the lab and into the wild: the influence of portion size on food intake in laboratory vs. real‐world settings. Appetite, 162, 105160. 33556391 10.1016/j.appet.2021.105160

[acer70115-bib-0022] Hardy, L. , Parker, S. , Hartley, L. & Hogarth, L. (2018) A concurrent pictorial drug choice task marks multiple risk factors in treatment‐engaged smokers and drinkers. Behavioural Pharmacology, 29(8), 716–725. 30169375 10.1097/FBP.0000000000000421

[acer70115-bib-0023] Hayes, M.H.S. & Patterson, D.G. (1921) Experimental development of the graphic rating method. Psychological Bulletin, 18, 98–99.

[acer70115-bib-0024] Hogarth, L. (2020) Addiction is driven by excessive goal‐directed drug choice under negative affect: translational critique of habit and compulsion theory. Neuropsychopharmacology, 45(5), 720–735. 31905368 10.1038/s41386-020-0600-8 PMC7265389

[acer70115-bib-0025] Hogarth, L. & Field, M. (2020) Relative expected value of drugs versus competing rewards underpins vulnerability to and recovery from addiction. Behavioural Brain Research, 394, 112815. Available from: 10.1016/j.bbr.2020.112815 32707138 PMC7495042

[acer70115-bib-0026] Jackson, A. , Stephens, D. & Duka, T. (2001) A low dose alcohol drug discrimination in social drinkers: relationship with subjective effects. Psychopharmacology, 157, 411–420. 11605101 10.1007/s002130100817

[acer70115-bib-0027] Jones, A. , Rose, A.K. , Cole, J. & Field, M. (2013) Effects of alcohol cues on craving and ad libitum alcohol consumption in social drinkers: the role of disinhibition. Journal of Experimental Psychopathology, 4(3), 239–249.

[acer70115-bib-0028] Klein, S.A. & Hilbig, B.E. (2019) On the lack of real consequences in consumer choice research. Experimental Psychology, 66, 68–76. 30777510 10.1027/1618-3169/a000420

[acer70115-bib-0029] Larsen, H. , Engels, R.C. , Granic, I. & Overbeek, G. (2009) An experimental study on imitation of alcohol consumption in same‐sex dyads. Alcohol and Alcoholism, 44(3), 250–255. 19240054 10.1093/alcalc/agp002

[acer70115-bib-0030] Larsen, H. , Engels, R.C. , Wiers, R.W. , Granic, I. & Spijkerman, R. (2012) Implicit and explicit alcohol cognitions and observed alcohol consumption: three studies in (semi) naturalistic drinking settings. Addiction, 107(8), 1420–1428. 22260335 10.1111/j.1360-0443.2012.03805.x

[acer70115-bib-0031] Larsen, H. , Overbeek, G. , Granic, I. & Engels, R.C. (2010) Imitation of alcohol consumption in same‐sex and other‐sex dyads. Alcohol and Alcoholism, 45(6), 557–562. 20847061 10.1093/alcalc/agq053

[acer70115-bib-0032] Levy, D.J. & Glimcher, P.W. (2012) The root of all value: a neural common currency for choice. Current Opinion in Neurobiology, 22(6), 1027–1038. Available from: 10.1016/j.conb.2012.06.001 22766486 PMC4093837

[acer70115-bib-0033] MacKillop, J. (2006) Factor structure of the alcohol urge questionnaire under neutral conditions and during a cue‐elicited urge state. Alcoholism: Clinical and Experimental Research, 30(8), 1315–1321. 16899034 10.1111/j.1530-0277.2006.00159.x

[acer70115-bib-0034] MacKillop, J. & Murphy, J.G. (2007) A behavioral economic measure of demand for alcohol predicts brief intervention outcomes. Drug and Alcohol Dependence, 89(2–3), 227–233. Available from: 10.1016/j.drugalcdep.2007.01.002 17289297

[acer70115-bib-0035] MacKillop, J. , Murphy, J.G. , Tidey, J.W. , Kahler, C.W. , Ray, L.A. & Bickel, W.K. (2009) Latent structure of facets of alcohol reinforcement from a behavioral economic demand curve. Psychopharmacology, 203, 33–40. 18925387 10.1007/s00213-008-1367-5 PMC2774887

[acer70115-bib-0036] Mair, P. & Wilcox, R. (2020) Robust statistical methods in R using the WRS2 package. Behavior Research Methods, 52, 464–488. 31152384 10.3758/s13428-019-01246-w

[acer70115-bib-0038] McGrath, E. , Jones, A. & Field, M. (2016) Acute stress increases ad‐libitum alcohol consumption in heavy drinkers, but not through impaired inhibitory control. Psychopharmacology, 233, 1227–1234. 26815361 10.1007/s00213-016-4205-1 PMC4801987

[acer70115-bib-0039] Merrill, J.E. & Aston, E.R. (2020) Alcohol demand assessed daily: validity, variability, and the influence of drinking‐related consequences. Drug and Alcohol Dependence, 208, 107838. 31954948 10.1016/j.drugalcdep.2020.107838 PMC7050944

[acer70115-bib-0040] Moeller, S.J. , Beebe‐Wang, N. , Woicik, P.A. , Konova, A.B. , Maloney, T. & Goldstein, R.Z. (2013) Choice to view cocaine images predicts concurrent and prospective drug use in cocaine addiction. Drug and Alcohol Dependence, 130(1–3), 178–185. 23218913 10.1016/j.drugalcdep.2012.11.001 PMC3609942

[acer70115-bib-0041] Monk, R.L. & Heim, D. (2013) A critical systematic review of alcohol‐related outcome expectancies. Substance Use & Misuse, 48(7), 539–557. 23647167 10.3109/10826084.2013.787097

[acer70115-bib-0042] Monk, R.L. & Heim, D. (2014) A real‐time examination of context effects on alcohol cognitions. Alcoholism: Clinical and Experimental Research, 38(9), 2454–2459. 25257294 10.1111/acer.12504

[acer70115-bib-0043] Motschman, C.A. , Amlung, M. & McCarthy, D.M. (2022) Alcohol demand as a predictor of drinking behavior in the natural environment. Addiction, 117(7), 1887–1896. 35112741 10.1111/add.15822 PMC10061588

[acer70115-bib-0045] Murphy, J.G. & MacKillop, J. (2006) Relative reinforcing efficacy of alcohol among college student drinkers. Experimental and Clinical Psychopharmacology, 14(2), 219–227. Available from: 10.1037/1064-1297.14.2.219 16756426

[acer70115-bib-0044] Murphy, J.G. , Dennhardt, A.A. , Martens, M.P. , Yurasek, A.M. , Skidmore, J.R. , MacKillop, J. et al. (2015) Behavioral economic predictors of brief alcohol intervention outcomes. Journal of Consulting and Clinical Psychology, 83(6), 1033–1043. Available from: 10.1037/ccp0000032 26167945 PMC4658255

[acer70115-bib-0046] Myers, C.E. , Interian, A. & Moustafa, A.A. (2022) A practical introduction to using the drift diffusion model of decision‐making in cognitive psychology, neuroscience, and health sciences. Frontiers in Psychology, 13, 1039172. 36571016 10.3389/fpsyg.2022.1039172 PMC9784241

[acer70115-bib-0047] Owens, M.M. , Murphy, C.M. & MacKillop, J. (2015) Initial development of a brief behavioral economic assessment of alcohol demand. Psychology of Consciousness: Theory, Research and Practice, 2(2), 144–152. 27135038 10.1037/cns0000056 PMC4845629

[acer70115-bib-0048] Patil, I. (2021) Visualizations with statistical details: the'ggstatsplot'approach. Journal of Open Source Software, 6(61), 3167.

[acer70115-bib-0049] Pronk, T. , van Deursen, D.S. , Beraha, E.M. , Larsen, H. & Wiers, R.W. (2015) Validation of the Amsterdam beverage picture set: a controlled picture set for cognitive bias measurement and modification paradigms. Alcoholism, Clinical and Experimental Research, 39(10), 2047–2055. Available from: 10.1111/acer.12853 26431117 PMC5054858

[acer70115-bib-0050] Public Health England . (2016) Health matters: harmful drinking and alcohol dependence . GOV.UK. Available from: https://www.gov.uk/government/publications/health‐matters‐harmful‐drinking‐and‐alcohol‐dependence/health‐matters‐harmful‐drinking‐and‐alcohol‐dependence [Accessed 20th September 2024].

[acer70115-bib-0051] Rangel, A. , Camerer, C. & Montague, P.R. (2008) A framework for studying the neurobiology of value‐based decision making. Nature Reviews Neuroscience, 9(7), 545–556. Available from: 10.1038/nrn2357 18545266 PMC4332708

[acer70115-bib-0052] Ratcliff, R. & McKoon, G. (2008) The diffusion decision model: theory and data for two‐choice decision tasks. Neural Computation, 20(4), 873–922. Available from: 10.1162/neco.2008.12-06-420 18085991 PMC2474742

[acer70115-bib-0053] Revelle, W. (2020) psych: procedures for psychological, psychometric, and personality research . R package version, 2(5).

[acer70115-bib-0054] Robinson, E. , Hardman, C.A. , Halford, J.C. & Jones, A. (2015) Eating under observation: a systematic review and meta‐analysis of the effect that heightened awareness of observation has on laboratory measured energy intake. The American Journal of Clinical Nutrition, 102(2), 324–337. 26178730 10.3945/ajcn.115.111195

[acer70115-bib-0055] Robinson, E. , Oldham, M. , Sharps, M. , Cunliffe, A. , Scott, J. , Clark, E. et al. (2016) Social imitation of alcohol consumption and ingratiation motives in young adults. Psychology of Addictive Behaviors, 30(4), 442–449. 27322802 10.1037/adb0000150 PMC4913807

[acer70115-bib-0071] Room, R. , Mäkelä, P. , Benegal, V. , Greenfield, T.K. , Hettige, S. , Tumwesigye, N.M. et al. (2012) Times to drink: cross‐cultural variations in drinking in the rhythm of the week. International Journal of Public Health, 57(1), 107–117. 21553132 10.1007/s00038-011-0259-3 PMC3272154

[acer70115-bib-0057] Rose, A.K. & Duka, T. (2006) Effects of dose and time on the ability of alcohol to prime social drinkers. Behavioural Pharmacology, 17(1), 61–70. 16377964 10.1097/01.fbp.0000189814.61802.92

[acer70115-bib-0056] Rose, A.K. , Brown, K. , Field, M. & Hogarth, L. (2013) The contributions of value‐based decision‐making and attentional bias to alcohol‐seeking following devaluation. Addiction, 108(7), 1241–1249. Available from: 10.1111/add.12152 23614520 PMC3746131

[acer70115-bib-0058] Rosseel, Y. (2012) Lavaan: an R package for structural equation modeling. Journal of Statistical Software, 48, 1–36.

[acer70115-bib-0059] Saunders, J.B. , Aasland, O.G. , Babor, T.F. , De La Fuente, J.R. & Grant, M. (1993) Development of the alcohol use disorders identification test (AUDIT): WHO collaborative project on early detection of persons with harmful alcohol consumption‐II. Addiction, 88, 791–804. 8329970 10.1111/j.1360-0443.1993.tb02093.x

[acer70115-bib-0060] Smith, J.L. & Mattick, R.P. (2018) Are there sex differences in the relationship between heavy alcohol use and disinhibition? A meta‐analysis.

[acer70115-bib-0061] Stanesby, O. , Labhart, F. , Dietze, P. , Wright, C.J. & Kuntsche, E. (2019) The contexts of heavy drinking: a systematic review of the combinations of context‐related factors associated with heavy drinking occasions. PLoS One, 14(7), e0218465. 31291261 10.1371/journal.pone.0218465 PMC6619678

[acer70115-bib-0063] Tucker, J.A. & Vuchinich, R.E. (2015) Efficient and final causes of alcohol consumption. Addiction, 110(9), 1429–1430. Available from: 10.1111/add.12983 26223172

[acer70115-bib-0062] Tucker, J.A. , Cheong, J. & Chandler, S.D. (2021) Shifts in behavioral allocation patterns as a natural recovery mechanism: postresolution expenditure patterns. Alcoholism, Clinical and Experimental Research, 45(6), 1304–1316. Available from: 10.1111/acer.14620 33885166 PMC8549063

[acer70115-bib-0069] Vuchinich, R.E. , Tucker, J.A. , Acuff, S.F. , Reed, D.D. , Buscemi, J. & Murphy, J.G. (2023) Matching, behavioral economics, and teleological behaviorism: Final cause analysis of substance use and health behavior. Journal of the Experimental Analysis of Behavior, 119(1), 240–258. 36541360 10.1002/jeab.815 PMC13294744

[acer70115-bib-0064] Weinsztok, S.C. , Reed, D.D. & Amlung, M. (2023) Identifying substitute activities for alcohol consumption: a preliminary analysis. Addiction Research & Theory, 31(3), 209–219. 37303833 10.1080/16066359.2022.2135704 PMC10254569

[acer70115-bib-0065] Wickham, H. , François, R. , Henry, L. , Müller, K. & Vaughan, D. (2023) dplyr: a grammar of data manipulation . R package version, 1(2).

[acer70115-bib-0066] World Health Organization . (2018) Global status report on alcohol and health 2018. Geneva: World Health Organization.

[acer70115-bib-0067] Yates, J. , Rose, A.K. & Jones, A. (2023) Attempts to influence the value of alcohol by manipulating social influence and context. Substance Use & Misuse, 58(8), 1053–1061. 37129011 10.1080/10826084.2023.2205532

[acer70115-bib-0068] Yücel, M. , Oldenhof, E. , Ahmed, S.H. , Belin, D. , Billieux, J. , Bowden‐Jones, H. et al. (2019) A transdiagnostic dimensional approach towards a neuropsychological assessment for addiction: an international Delphi consensus study. Addiction, 114(6), 1095–1109. 30133930 10.1111/add.14424 PMC6386631

