# Supplementary material for: Social influence increases the value and consumption of alcohol in the laboratory
Source: Alcohol Clin Exp Res (Hoboken). 2025 Aug 11;49(9):2038–48. doi: 10.1111/acer.70115 (PMC12463762; doi:10.1111/acer.70115)

Supplementary materials

*Ad-libitum* sensitivity analyses

Male only

A 2 x 2 between subjects ANOVA was conducted to determine the effect of social influence and environment on the amount of alcohol consumed *ad-libitum*. There was no significant main effect of social influence on alcohol consumed (*F(*1,45) = 0.28, *p* =.600, *ηp^2 =^* .01). no significant main effect of environment (*F(*1,45) = 3.02, *p* =.089, *ηp^2 =^* .06) and no significant interaction (*F(*1,45) = 1.52, *p* =.224, *ηp^2 =^* .03).

Female only

A 2 x 2 between subjects ANOVA was conducted to determine the effect of social influence and environment on the amount of alcohol consumed *ad-libitum.* There was a significant main effect of social influence on alcohol consumed (*F*(1,84) = 12.96, *p*<.001, *ηp^2^* =.13) with more alcohol consumed in the positive appraisal condition (Mean =197.78, SD =114.27) compared to the confederate negative appraisal condition (Mean =122.73, SD =77.16). However, there was no significant effect of environment ((*F*(2,84) = 0.67, *p* =.516, *ηp^2^* =.02) or interaction (*F*(1, 84) = 0.54, *p* = .466, *ηp²* = .01).

Matched participant and confederate gender

A 2 x 2 between subjects ANOVA was conducted to determine the effect of social influence and environment on the amount of alcohol consumed *ad-libitum.* There was no significant main effect of social influence on alcohol consumed (*F(*1,64) = 1.07, *p* =.305, *ηp^2 =^* .012). no significant main effect of environment (*F(*2,64) = 2.93, *p* =.061, *ηp^2 =^* .08) and no significant interaction (*F(*1,64) = 0.79, *p* =.079 , *ηp^2 =^* .01).

Opposite participant and confederate gender

A 2 x 2 between subjects ANOVA was conducted to determine the effect of social influence and environment on the amount of alcohol consumed *ad-libitum.* There was a significant main effect of social influence on alcohol consumed (*F*(1,66) = 8.87, *p*=.004, *ηp^2^* =.12) with more alcohol consumed in the positive appraisal condition (Mean =244.58, SD =123.72) compared to the confederate negative appraisal condition (Mean =166.73, SD =96.39). However, there was no significant effect of environment ((*F*(1,66) = 1.17, *p* =.283, *ηp^2^* =.02) or interaction (*F*(1, 66) = 2.63, *p*=.110, *ηp²* = .04).

Extra factor of matched status

A 2x2x2 between subjects ANOVA was conducted to determine the effect of social influence, environment and participant and confederate gender matched status on the amount of alcohol consumed *ad-libitum*. There was a significant main effect of social influence on alcohol consumed (*F*(1,131) = 7.88, *p*=.006, *ηp^2^* =.06) with more alcohol consumed in the positive appraisal condition (Mean =244.58, SD =123.72) compared to the confederate negative appraisal condition (Mean =166.73, SD =96.39). There were no significant main effects of environment (*F*(1,131) = 1.35, *p*=.247, *ηp^2^* =.01) or matched gender status (*F*(1,110) = 1.09, *p*=.298, *ηp^2^* =.01). There were also no significant interactions between social influence and environment (*F*(1,131) = 2.53, *p*=.114, *ηp^2^* =.02), social influence and matched status (*F*(1,131) = 2.37, *p*=.126, *ηp^2^* =.2) and social influence, environment and matched status (*F*(1,131) = 0.13, *p*=.724, *ηp^2^* =.00). There was however a significant interaction between environment and matched gender status (*F*(1,131) = 5.10, *p*=.026, *ηp^2^* =.04).

Table shows post-hoc pairwise contrasts results across environment and matched status

| **Table:** Post-hoc pairwise contrasts results across environment and matched gender status with *ad-libitum* means (±SD) | | | | | |
| --- | --- | --- | --- | --- | --- |
|  | M (±SD) | Bar Lab/ Unmatched | Standard Lab/ Unmatched | Bar Lab/ Matched | Standard Lab/ Matched |
| Bar Lab/ Unmatched | 220.00 (120.91) |  | *t* (131) =1.04, *p=1.000,*  *d*=0.19 | *t* (131) =2.45, *p=.093*  *d* =0.34 | *t*(131) =.0.24 *p=1.000* *d* =0.03 |
| Standard Lab/ Unmatched | 242.38 (115.44) |  |  | *t*(131) =1.58 *p=1.000*  *d* =0.23 | *t*(131) =0.77, *p=1.000* *d* =0.11 |
|  |  |  |  |  |  |
| Bar Lab/ Matched | 282.86 (112.28) |  |  |  | *t* (131) =2.16, *p* =.196 *d* =0.29 |
| Standard Lab/ Matched | 222.59 (132.33) |  |  |  |  |

**Table** shows no significant differences between environment and matched gender status contrasts

*Discussion of sensitivity analyses*

Exploratory analyses revealed that gender may moderate the influence of social and environmental factors on alcohol consumption. For instance, male participants appeared to consume more in the non-bar setting, and women were more influenced by positive appraisals. While these effects did not consistently reach statistical significance, they suggest that gender-specific dynamics merit further investigation in larger samples particularly as the study was only powered to detect main effects. Therefore, it may have been underpowered to detect complex interactions.

SIS

Paired samples t-tests were conducted to determine any difference in the Subjective Intoxication Scale ratings

*SIS Lightheaded*

A paired samples t-test was conducted to determine a difference in SIS lightheaded between time 1 pre-drink and time 2 post-drink. There was a significant difference in SIS lightheaded time 1 compared with time 2 (t (136) = 2.58, p =.005) with greater SIS lightheaded rating in time 2 (Mean = 19.81 SD = 21.55) than time 1 (Mean = 15.59 SD = 20.60).

*SIS Irritable*

A paired samples t-test was conducted to determine a difference in SIS irritable between time 1 pre-drink and time 2 post-drink. There was no significant difference in SIS irritable between time 1 and time 2 (t (136) = 0.52, p =.302).

*SIS Stimulated*

A paired samples t-test was conducted to determine a difference in SIS stimulated between time 1 pre-drink and time 2 post-drink. There was a significant difference in SIS stimulated time 1 compared with time 2 (t (136) = 4.52, p<.001) with greater SIS stimulated rating in time 2 (Mean = 41.47 SD = 24.53) than time 1 (Mean = 32.23 SD = 23.81).

*SIS Alert*

A paired samples t-test was conducted to determine a difference in SIS alert between time 1 pre-drink and time 2 post-drink. There was no significant difference in SIS alert between time 1 and time 2 (t (136) = 0.88, p =.191).

*SIS Relaxed*

A paired samples t-test was conducted to determine a difference in SIS relaxed between time 1 pre-drink and time 2 post-drink. There was a significant difference in SIS relaxed time 1 compared with time 2 (t (136) = 1.66, p=.049) with greater SIS relaxed rating in time 2 (Mean = 69.08 SD = 21.88) than time 1 (Mean = 65.87 SD = 24.53).

*SIS Contented*

A paired samples t-test was conducted to determine a difference in SIS contented between time 1 pre-drink and time 2 post-drink. There was a significant difference in SIS contented time 1 compared with time 2 (t (136) = 1.88, p=.031) with greater SIS contented rating in time 2 (Mean = 71.50 SD = 19.13) than time 1 (Mean = 68.93 SD = 19.69).

Overall, we found that participants were significantly more lightheaded, stimulated, relaxed and contented in time 2 compared to time 1

*Lab Photos*

Figure 2: Bar Lab


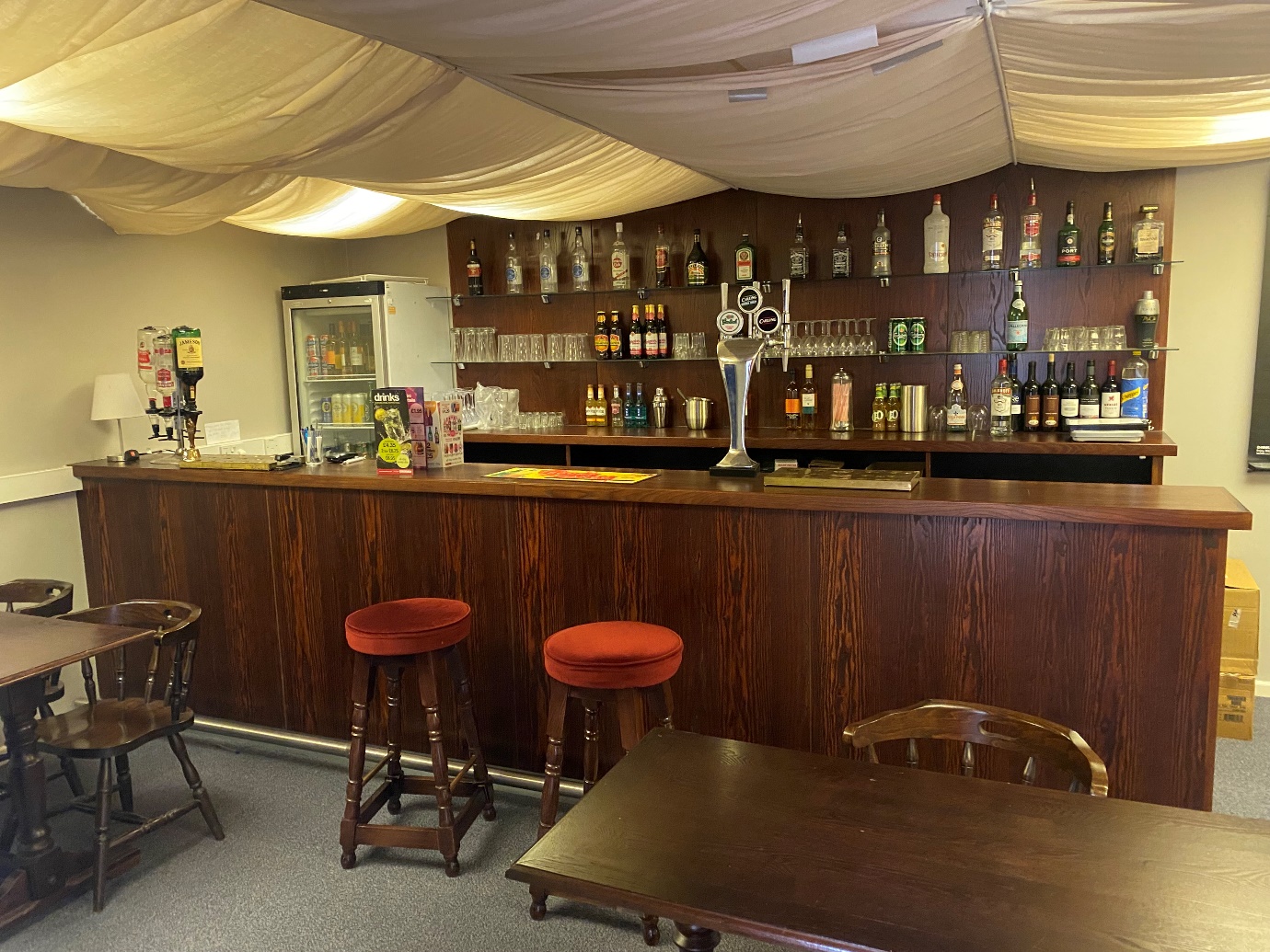


Figure 1: Standard Unadorned Lab
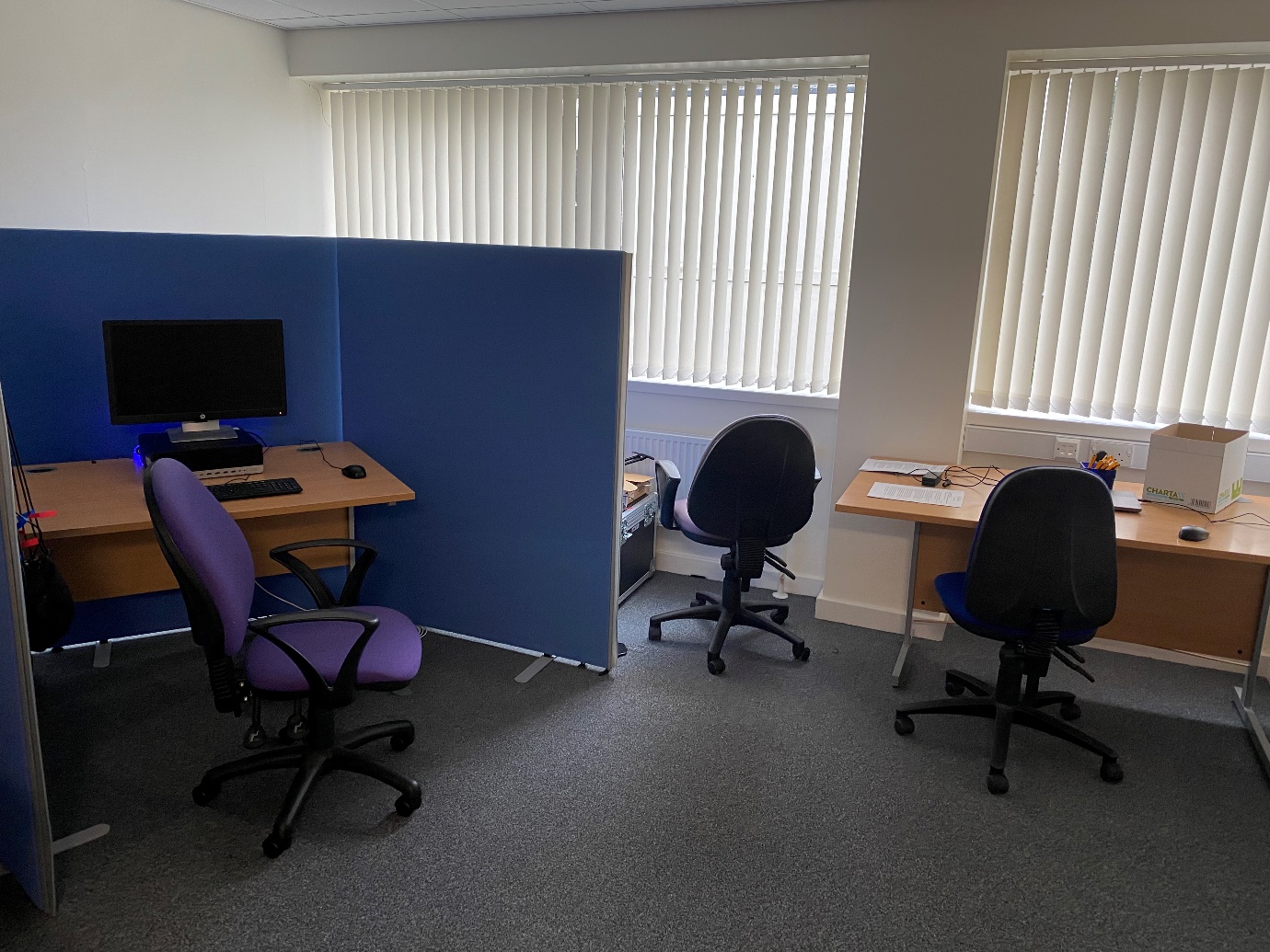

Supplement: Supplementary file 1 — Appendix S1 [file ACER-49-2038-s001.docx]
